# Study of diffuse scattering on facial surface using ray tracing approach

**DOI:** 10.1038/s41598-025-89113-x

**Published:** 2025-04-12

**Authors:** Po-Yen Lai, Ray Jia Hong Ng, Tatsuya Omotezako, Huizhe Liu, Wenjun Ding, Lim Jiun Yeu, Akira Matsubara

**Affiliations:** 1 Institute of High Performance Computing (IHPC), Agency for Science Technology and Research (A*STAR), 1 Fusionopolis Way, #16-16 Connexis, Singapore, 138632 Republic of Singapore; 2 Procter & Gamble Singapore Innovation Center, 70 Biopolis Street, Singapore, 138547 Republic of Singapore; 3 Kobe Innovation Center, Procter & Gamble Innovation GK, Kobe, Japan

**Keywords:** Skin manifestations, Applied optics

## Abstract

This study investigates the role of topographic attributes in light scattering and diffuse reflection on the skin surface, and diffuse transmission across the surface layer. Validated ray-tracing simulations establish a quantitative link between subvisible micro texture (SMT) and macro texture to skin optical properties, representing youthful and healthy skin characteristics. Our findings reveal the dominant role of the subvisible micro texture parameter (${u}_{w}/{u}_{h}$, the ratio of width to height) in governing light scattering. Smaller ratios, corresponding to fine SMT, result in increased diffuse reflection and create a more pronounced soft-focus effect, which plays a critical role in driving profound skin appearance such as radiance. While the macro texture parameter also influences scattering, its impact is less significant. Additionally, we quantify the impact of SMT on the transversal light movement from inside the skin to outside it. This simulation showed that more incident light leaves after traveling inside skin with smaller ${u}_{w}/{u}_{h}$, a measure of finer SMT. Our validated 2D subvisible micro texture model accurately computes the light-skin interactions influenced by the various levels of the skin’s topographic features, offering valuable insights for cosmetics, dermatology, and aesthetic medical imaging.

## Introduction

The optical properties of human skin are pivotal in various fields, including dermatology, cosmetic science, and aesthetic medical imaging. These properties are influenced by the complex interplay of light with the skin’s surface and subsurface structures. Light interaction with the skin involves multiple processes, including absorption, reflection, and scattering, which collectively determine the visual perception of skin appearance^1–5^. Understanding these interactions is essential for developing accurate models and improving diagnostic and therapeutic techniques.

Surface topography is known to play a crucial role in regulating the surface and subsurface optical properties of materials, which in turn affect their appearance. Through the detail observation of topographic attributes on skin surface, the existence of various scales of structures on the skin surface has been reported^6,7^. Topographies composed of crista cutis (skin ridges) and sulcus cutis (skin furrows) are visible structure recognized as fine line texture on skin surface by naked eye. Additionally, unique topographical attributes have been observed on the surface of crista cutis and corneocyte cells^6–8^, described as the 1st-4th relief^6,7^. Recently, it has been proposed that these subvisible micro textures (SMT) play a central role in regulating light scattering on the skin surface, contributing to visible skin appearances such as softness, radiance, and smoothness, even though these structures themselves are invisible to the naked eye^5,9^. However, our understanding of how these macro-textures and SMT directly interact with incident light to regulate light scattering on the skin surface is still limited.

On the other hand, subsurface scattering, also known as diffuse transmission, involves light penetrating the skin, interacting with various skin layers, and re-emerging at different points, contributing to the skin’s translucent appearance^6,10,11^. The translucency of the skin, enhanced by diffuse transmission, plays a significant role in the visual perception of skin health and beauty, as it helps diminish imperfections and create a more natural and youthful look^12–14^. However, there is limited experimental evidence to understand how skin surface topography contributes to light translucency by regulating light transmission and diffusion on the skin surface.

Closing this knowledge gap is crucial for fields like cosmetics, dermatology, and aesthetic medical imaging. Accurate models of how light interacts with skin can improve diagnostic accuracy, treatment planning, and product development in these areas. Additionally, Monte Carlo Multi-Layered (MCML) simulations^10,11,15–18^ have been valuable for understanding light propagation within skin tissue.

However, these simulations often rely on overly simplified models without considering skin surface topography or with visible macro-topography. Therefore, incorporating a more accurate representation of skin topography SMT as well as macro-topography could significantly enhance the accuracy of MCML simulations.

Building on these findings, the current study investigates how SMT affects optical scattering, focusing on backscattering and forward scattering. We use a detailed workflow involving laser microscopy, parameterization, and ray-tracing simulations to model optical scattering. Our goal is to develop a computational model that quantifies light scattering based on skin surface structures and derive empirical formulas relating these structures to the scattering intensity and distribution. This study provides a quantitative understanding of how SMT impacts both diffuse reflection and transmission, elucidating its influence on the soft-focus effect and translucency. By developing and validating an equivalent 2D SMT model, we offer a predictive tool for light scattering based on skin surface features.

In this study, we present a comprehensive approach to analyze light scattering on facial skin surfaces. Utilizing a physically accurate and geometrically flexible ray-tracing (RT) method, we categorize surface scattering into diffuse reflection and transmission. The detailed methodology, outlined in the Methods section, involves preparing skin replicas and polymethyl methacrylate (PMMA) plates, scanning surfaces using laser microscopy, and parameterizing surface structures to establish equivalent 2D SMT models. We conduct a parametric study of optical scattering using Monte Carlo forward ray-tracing, simulating light scattering on various SMT structures. The results of these simulations are summarized, revealing the relationship between surface radiation scattering and SMT structure. We then delve into detailed characterizations of diffuse reflection and transmission from different skin samples, examining the influence of SMT parameters through a comprehensive parametric study. The implications of SMT on the soft-focus effect and skin translucency are also discussed. Finally, we conclude with a discussion and summary of our key findings.

## Methods

### Subvisible micro texture (SMT) scattering and methodological workflow

**Fig. 1:**
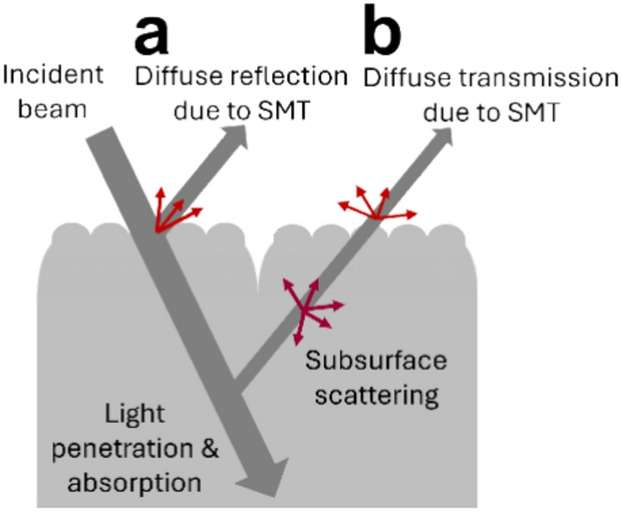
Schematic illustration of optical scattering behaviors investigated in this study: (**a**) diffuse reflection, (**b**) diffuse transmission due to skin micro texture.

To investigate the variations in optical scattering induced by subvisible micro texture (SMT) as well as macro texture, surface scattering can be categorized into diffuse reflection and transmission. As illustrated in Fig. 1(a), diffuse reflection occurs when light is incident on the skin surface and scattered back into the same medium (air). This mechanism is directly related to the soft-focus effect of SMT. Figure 1(b) depicts diffuse transmission, which happens when light penetrates the skin, undergoes subsurface scattering, and is diffusely transmitted back into the air in the presence of SMT. In real-world scenarios, subsurface scattering involves light interacting with various skin microstructures, such as collagen fibers and blood vessels scattering the light, and chromophores absorbing it^12^, which is out of the scope of this study. This mechanism directly affects the skin’s translucency. To quantitatively evaluate the impact of SMT in these two scattering scenarios, this study examines diffuse reflection by illuminating the skin surface from a specific direction in air, whereas for diffuse transmission, it considers light traveling in various directions within the skin model. This mimics light sources from subsurface scattering, allowing the light to interact with the SMT and transmit through the air.

**Fig. 2:**
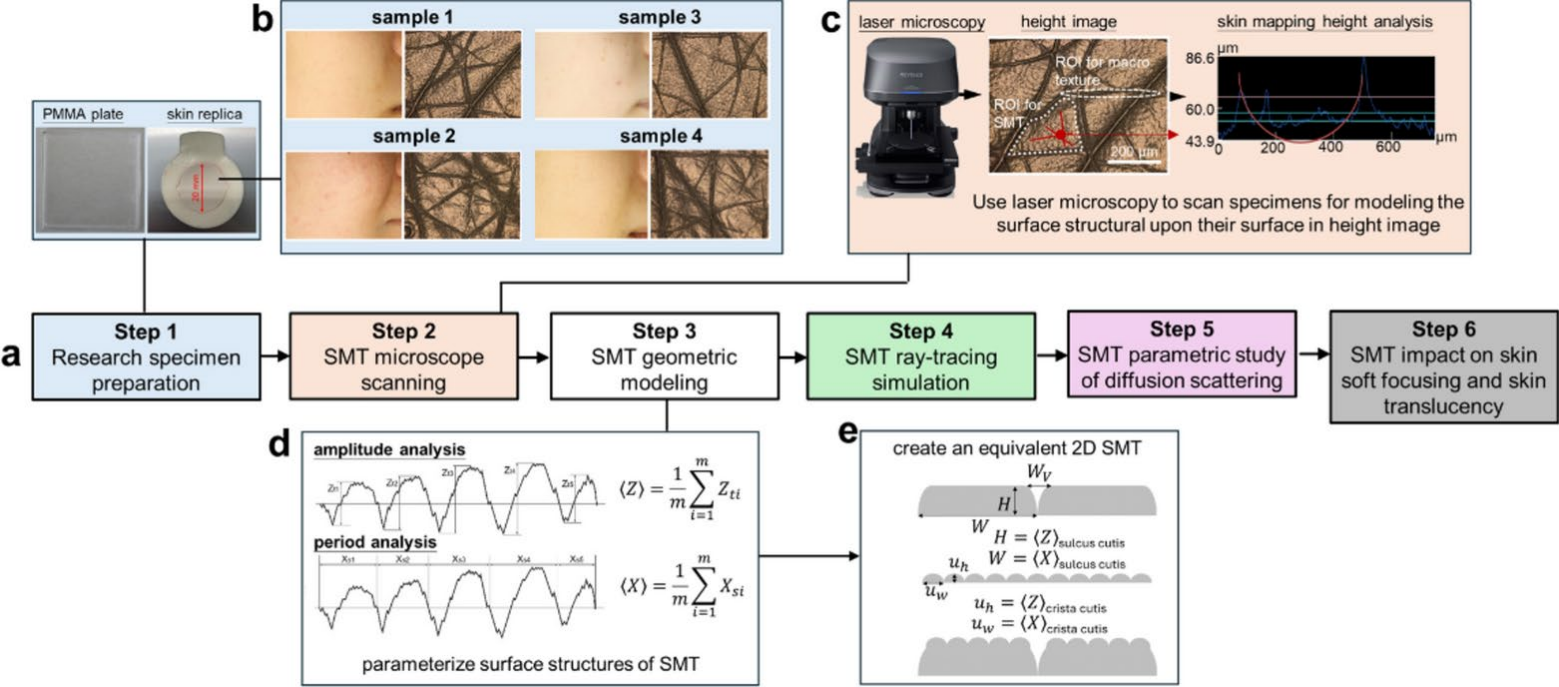
illustrates the workflow of this study: (**a**) A flowchart outlining the research steps. (**b**) The study specimens, including a PMMA plate and skin replicas. The inset on the right shows 4 representative skin specimens and their SMT scan. (**c**) The procedures of SMT scans, obtained using a 3D laser scanning microscope (VK-X300, KEYENCE CORPORATION, Japan), and analysis. Middle picture illustrates the Regions of Interest (ROIs) for SMT (indicated by the white dotted line) and macro texture indicated by the white dashed line). The red line represents the direction of the microscopic scan. (**d**) Parameterization (amplitude and period) of the scanned SMT maps. (**e**) Construction of equivalent 2D SMT models.

Figure 2(a) outlines the study’s 6-step workflow.

*Step 1*: *Research specimen preparation:* As depicted in Fig. 2(b), the specimens for this study were categorized into two types: skin replicas and polymethyl methacrylate (PMMA) plates. Skin replicas were collected from the facial skin of healthy East Asian females using Silflo silicone polymers (Monaderm, Monaco). There were consist of a total of 60 specimens, with 4 representative examples shown in the figure. The R120 Replica Ring Locator (Clinical & Derm, USA) was used to apply the polymer onto a 19-mm diameter area of the lower cheek. Helioplate PMMA plates (Helioscreen, Belgium), *i.e.,*molded PMMA plates (HD6) and sandblasted PMMA plates (SB6), were employed in this study for benchmarking purposes, as their surface topography resembles that of skin^13^.

*Step 2*: *Skin topography microscope scanning*: As depicted in Fig. 2(c), A 3D laser scanning microscope (VK-X300, Keyence, Japan) was employed to scan a 708 μm × 531 μm area of the replica surface. The acquired topography data (height image) was then analyzed using the provided software VK Analyzer ver. 4.0.0.0 (Keyence Corporation, Japan).

*Step 3*: *Skin topography geometric modeling*: Fig. 2(d) illustrates the process of parameterizing the skin surface from the topography image obtained in *Step 2* for simulation studies. The image showcasing subvisible micro textures (central panel) from Fig. 2(c) is segmented into regions representing macro texture (sulcus cutis, delineated by the white dashed line) and subvisible micro texture areas (SMT, marked by white dashed outlines). Multiple regions of interest (ROIs) are strategically chosen to assess both macroscopic features and the ridge surface morphology, the latter exhibiting a periodicity on the order of hundreds of μm. Solid red lines denote cross-sections of the subvisible micro texture regions within these ROIs, which are subsequently analyzed to determine height and period parameters, typically on the order of several micrometers and tens of μm, respectively. The SMT structure is characterized by averaging data extracted from each ROI.

To simplify the SMT, as shown in Fig. 2(e), we analyzed multiple samples and approximated the macro skin structure as a geometric shape with a flat top and steep edges, representing the sulcus cutis. The average spatial periodicity is denoted as $W$, the average height (depth) as $H$, and the average width of the sulcus cutis as ${W}_{V}$. The macro texture’s edges are modeled by an elliptical function with height $H$ and major axis ${W}_{V}$, while the SMT is represented by an elliptical function with average height ${u}_{h}$ and period ${u}_{w}$. This combination yields a 2D geometric model suitable for quantitative parametric studies. 

Table 1 summarizes the surface structure parameters of all research objects (including 4 representative skin replicas and 2 PMMA plates) in this study, obtained using the methods described in Fig. 2(d) and Fig. 2(e). The last column defines a parametric space for our study, encompassing 60 distinct skin replicas and providing the maximum and minimum values for each parameter.

**Table 1:** SMT parameters for all specimens in this study were obtained using the method described in Fig. 2(d) and Fig. 2(e).

	Specimen	$H$[μm]	$W$[μm]	${W}_{V}$[μm]	${u}_{h}$[μm]	${u}_{w}$[μm]
PMMA (*n* = 1.49)	HD6	-	-	-	18.85	256.4
	SB6	20.0	200.0	20.0	2.0	20.0
Skin replica (*n* = 1.40)	sample 1	29.7	288.2	40.0	2.7	32.5
	sample 2	17.9	240.8	40.0	2.1	53.4
	sample 3	18.1	286.6	40.0	2.0	30.7
	sample 4	26.0	264.3	40.0	2.7	58.4
	min to max across 60 samples	7.5–56.6	103–395.5	20–60	1.5–3.2	30–58.4

The skin ensemble consists of 60 different skin specimens and includes the parametric space along with the possible range (from minimum to maximum) for parametric study. PMMA parameters were only used for benchmarking purposes. In simulations, The refractive index of PMMA and skin are set to 1.49 and 1.40, respectively.

*Step 4*: *Skin topography ray-tracing simulation*:

Based on *Step 3*, we can rapidly generate equivalent skin models by modeling various SMT structures with different parameters. These models can then be used to simulate diffuse reflection and transmission properties. For simulations, this study extends the 2D equivalent skin model to 3D, assuming no variation along the extra axis, which can significantly reduce the computational time. The Monte Carlo forward ray-tracing technique, implemented in Zemax OpticStudio^14^, is used to trace rays from each mesh point to the light source, simulating optical scattering. This technique has previously been validated for simulating irradiance received by complex surfaces^19^.

*Step 5*: *Skin topography parametric study of diffusion scattering*:

The scattering behavior of light is primarily defined by the radiation angular distribution ($\theta$), which follows a Gaussian distribution characterized by its intensity $A$ and diffuse broadening width $\sigma$:

1 $$A{e}^{-\frac{{\left(\theta -\mu \right)}^{2}}{2{\sigma }^{2}}}$$

where, $\mu$ is the central angle of the scattering profile. The scattering radiation angular distribution obtained from ray-tracing simulations for various SMT can be fit using **Eq. **1. The fitting parameters ($A$ and $\sigma$) are analyzed for their correlation with the SMT parameters established in *Step 3*. Adopting Spearman’s rank correlation test, we identify the SMT parameters most significantly correlated with $A$ and $\sigma$, and subsequently derive empirical formulas to quantify these relationships.

*Step 6*: *SMT impact on skin soft-focus effect and skin translucency*: Utilizing the simulation results from *Step 4* and the analysis of parametric study from *Step 5*, we summarize the impact of varying SMT structures on the skin soft-focus effect and translucency, subsequently comparing our findings with previous observations^20^.

### Benchmark validation of proposed model

**Fig. 3:**
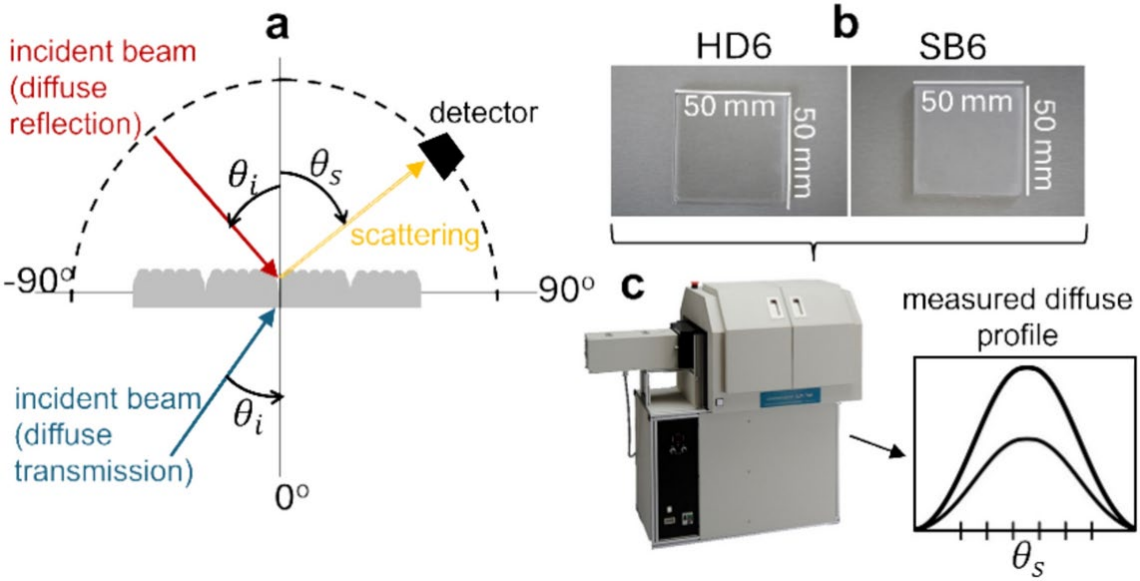
Experimental setup for benchmark measurements for both diffuse reflection and transmission. (**a**) Schematic of the physical system, illustrating the incident light paths (red arrow: diffuse reflection, blue arrow: diffuse transmission) at an incident angle (${\theta }_{i}$), scattered light (yellow arrow) at a scattering angle (${\theta }_{s}$), and detector orientation (zenith direction, ± 90° scattering plane with azimuthal symmetry). (**b**) PMMA plate samples. (**c**) Gonio Photometer GP-700 used for light scattering measurements.

To validate the accuracy of our simulation results, we conducted a series of benchmark experiments. Figure 3 illustrates the experimental setup, including a schematic of the physical system with incident light paths (red arrow: diffuse reflection, blue arrow: diffuse transmission), scattered light (yellow arrow), and detector orientation (zenith direction, ± 90° scattering plane with azimuthal symmetry) in **(a)**, PMMA plate samples in **(b)**, and the GP-700 Gonio Photometer (Murakami Color Research Laboratory, Japan) used for light scattering measurements in **(c)**.

**Fig. 4:**
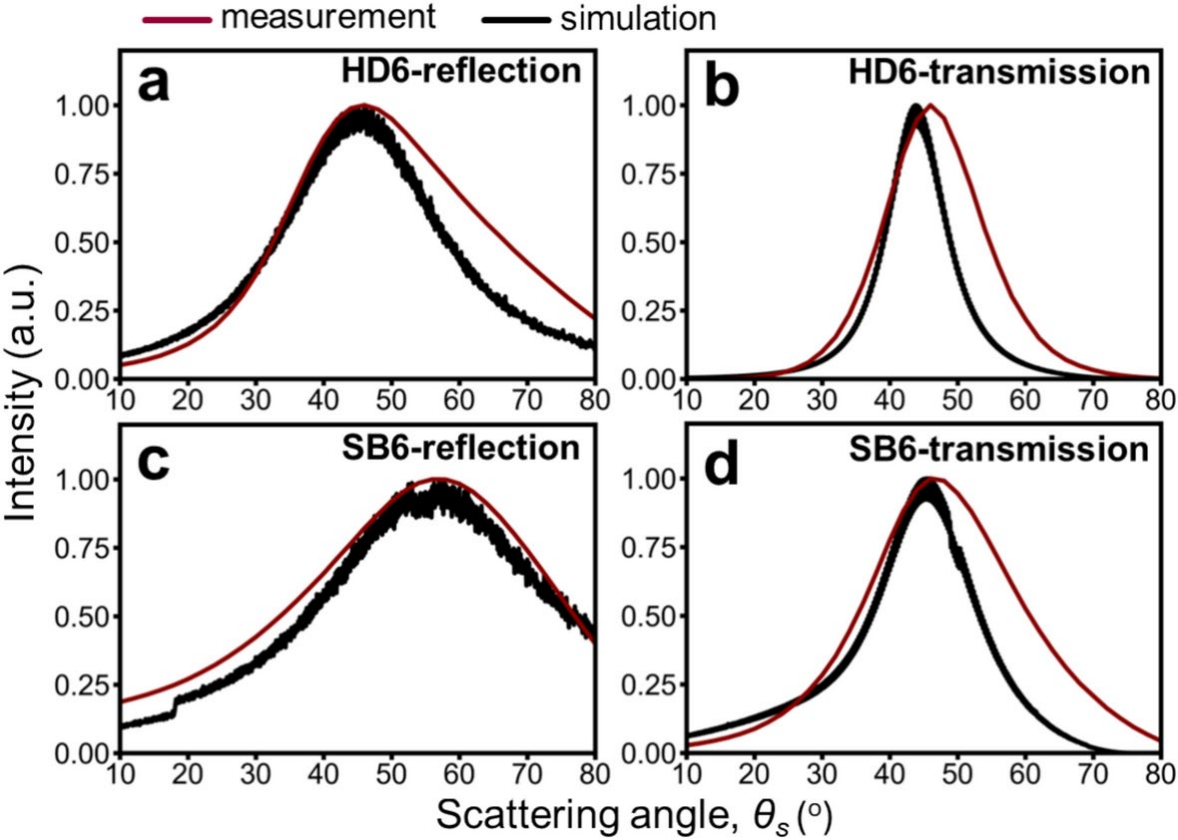
Benchmarking results for diffuse reflection and transmission of PMMA HD6 and SB6 (refractive index of 1.49). Red lines represent experimental measurements, while black lines represent simulations based on 2D geometric skin models. (**a**) Diffuse reflection intensity distribution versus scattering angle for PMMA HD6. (**b**) Diffuse transmission intensity distribution versus scattering angle for PMMA HD6. (**c**) Diffuse reflection intensity distribution versus scattering angle for PMMA SB6. (**d**) Diffuse transmission intensity distribution versus scattering angle for PMMA SB6.

Figure 4 compares diffuse reflection and transmission scattering in terms of radiation angular distributions for experimental measurements and simulations of PMMA HD6 and SB6 (refractive index = 1.49). The angle between the incident light and the object plane normal is 45°. Ray-tracing simulations based on 2D geometric skin models are compared to experimental data for both PMMA types (HD6 in **(a)** and **(b)**, SB6 in **(c)** and **(d)**).

Measurements show SB6 scatters light more diffusely than HD6, for both reflection and transmission scattering. Additionally, reflection scattering is consistently more diffuse than transmission scattering for both PMMA types. The 2D geometric model qualitatively supports these findings. The limited selection range (incident beam size significantly larger than the extracted area for scanning) introduces slight discrepancies between measurements and simulations. These discrepancies are more pronounced for diffuse transmission due to the simplification in the simulations, which assume light propagation through a uniform medium and neglect subsurface scattering within the PMMA material. Despite these limitations, the results obtained from two methods are consistent.

Furthermore, HD6 and SB6 exhibit differences in their SMT parameters (Table 1). Notably, HD6 lacks larger macro features (similar to the sulcus cutis) and has larger SMT (akin to the crista cutis) compared to SB6, which more closely resembles the SMT features of real skin. Despite these differences, both HD6 and SB6 can induce light scattering diffusion. This suggests that the SMT (${u}_{w}$ and ${u}_{h}$) plays a more significant role in light scattering diffusion, while the macro texture parameters ($W$*, *${W}_{V}$*,* and $H$) appear to have a less pronounced impact. We will further explore the relationship between each parameter and the level of scattering diffusion through a parametric study in the **Results** section, aiming to establish a quantitative description.

## Results

### Diffuse reflection from subvisible micro texture (SMT)

**Fig. 5:**
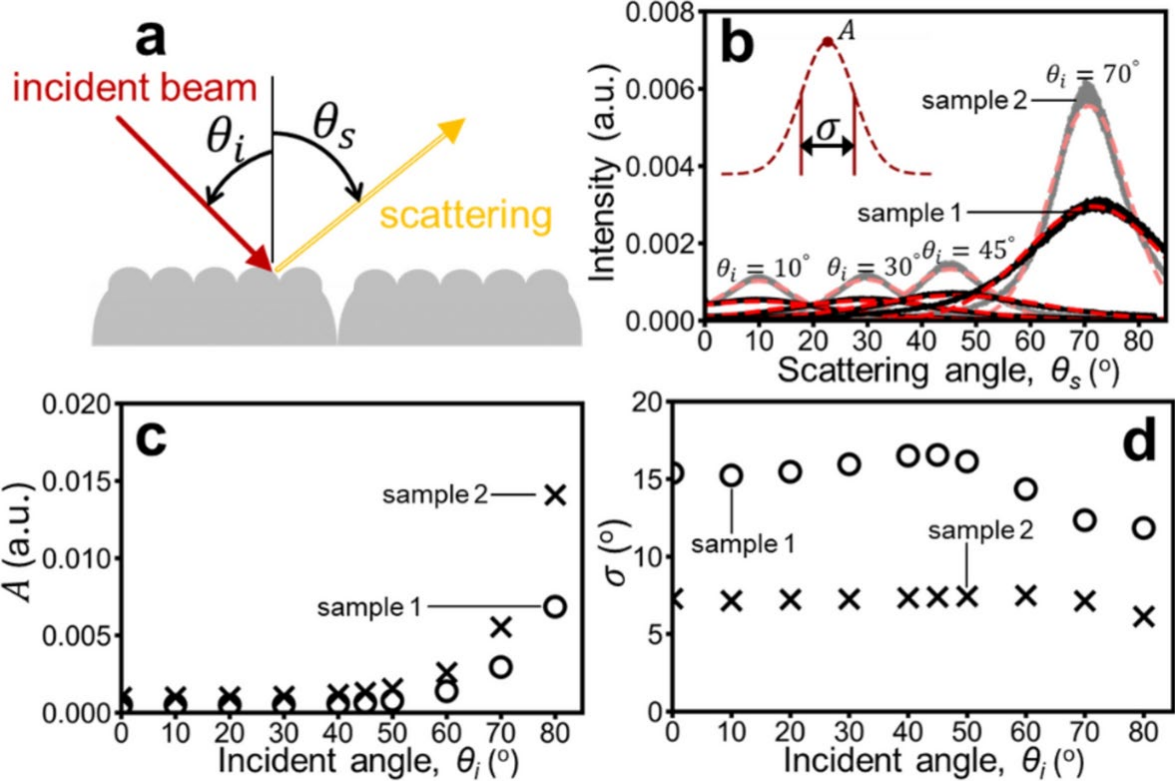
(**a**) Schematic illustration of the diffuse reflection process. (**b**) Comparison of scattering intensity distributions versus ${\theta }_{s}$ (scattering angle) for sample 1 (black) and sample 2 (gray). Solid lines represent simulated results, while dashed lines represent Gaussian fits. (**c**) Relationship between the Gaussian fit coefficient $A$ (intensity) and ${\theta }_{i}$(incident angle). (**d**) Relationship between the Gaussian fit coefficient $\sigma$ (width) and ${\theta }_{i}$(incident angle).

Figure 5(a) illustrates the diffuse reflection process, where incident light on the SMT is partially scattered. The refractive index of skin is set to 1.4 for the optical property analysis. This scattering behavior, centered around the specular reflection angle, is well-approximated by a Gaussian function (**Eq. **1) with intensity ($A$) and diffuse broadening width ($\sigma$) as key parameters. A larger $\sigma$implies wider angular spread and higher diffusion. Simulations were performed on samples 1 (visually softer skin^20^) and 2 (visually less soft skin^20^) from .

Table 1. Figure 5(b) compares their SMT-induced scattering in scattering angle space (${\theta }_{s}$) at various incident angles (${\theta }_{i}$). Sample 1 consistently shows broader scattering, though with lower peak intensity, than sample 2. Figure 5(c) and (d) present $A$ and $\sigma$ from Gaussian fits for ${\theta }_{i}=0$ to 90°. While $A$ increases with incident angle, $\sigma$ remains relatively constant. Notably, sample 1 consistently demonstrates more scattering diffusion than sample 2, regardless of ${\theta }_{i}$.

To identify SMT parameters directly correlated with $A$ and $\sigma$, Spearman’s rank correlation test was employed. We referenced Crista cutis Surface Reflection (CSR) and soft appearance parameters, measured from 60 skin samples in a previous work^20^, and analyzed their correlations with SMT parameters. CSR is defined as the average reflection intensity within the region of interest (ROI), while soft appearance is visually assessed based on the grade of tenderness and softness (see^20^ for details). Correlation results for the measured 60 skin samples are summarized in the first two columns of Table 2. Simulations, involving varying SMT (${u}_{w}$ and ${u}_{h}$) and macro texture ($W$*, *${W}_{V}$*,* and $H$) parameters for 19 samples under ${\theta }_{i}=$ 45°, yielded correlations with $A$ and $\sigma$, summarized in the last two columns of Table 2. Notably, CSR and soft appearance showed statistically significant correlations only with ${u}_{w}$, while in simulations, both $A$ and $\sigma$ correlated significantly with ${u}_{w}$ and ${u}_{h}$. CSR and $A$ exhibited positive correlations with ${u}_{w}$, while soft appearance and $\sigma$ showed a negative correlation. Simulation-based correlation tests revealed that compound parameters (${u}_{w}/{u}_{h}$, ${W}_{v}/W$) exhibited stronger correlations than individual SMT parameters. These dimensionless compound parameters also effectively reduce the parameter space. Although ${W}_{v}/W$ did not reach strict statistical significance (*p*-value = 0.065), its proximity to 0.05 justifies its use as a representative parameter for macro texture. As such, we selected ${u}_{w}/{u}_{h}$ as the representative parameter for SMT and ${W}_{v}/W$ for macro texture for subsequent parametric studies.

**Table 2:** Spearman’s rank correlation coefficients between SMT parameters and (left two columns) measured Crista cutis Surface Reflection (CSR) and soft appearance, and (right two columns) simulated Gaussian fit parameters $A$(intensity) and $\sigma$ (width).

Parameter	Measurement (N = 60)		Simulation (N = 19)	
	CSR	Soft appearance	$A$	$\sigma$
$W$	0.17	−0.08	0.41	−0.41
$H$	0.13	0.04	0.21	−0.33
${W}_{V}$			−0.18	0.15
${u}_{w}$	0.56***	−0.45***	0.74***	−0.73***
${u}_{h}$	0.02	−0.20	−0.55*	0.58**
${u}_{h}/{u}_{w}$	0.41***	−0.16	0.93***	−0.96***
${W}_{V}/H$			−0.26	0.34
$H/W$			−0.15	0.067
${W}_{V}/W$			−0.43	0.40

Significance levels are indicated by asterisks: *p < 0.05, **p < 0.01, ***p < 0.001.

**Fig. 6:**
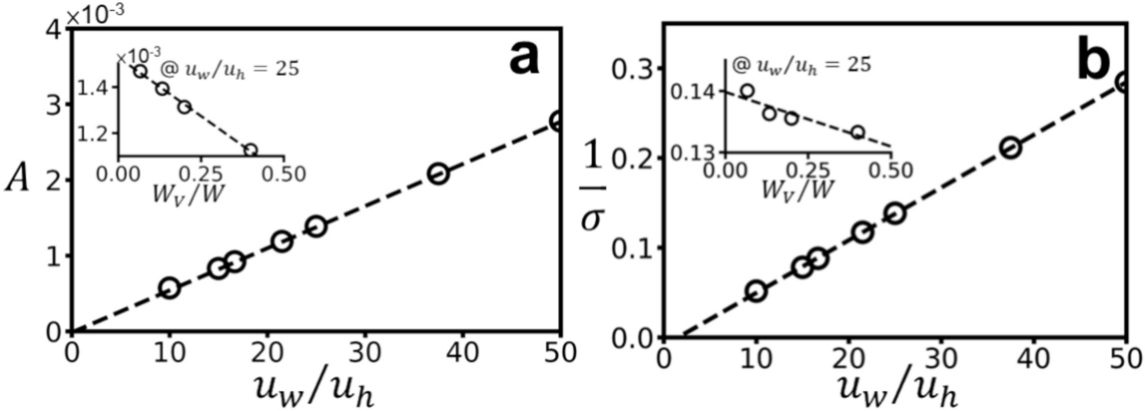
Linear relationships between Gaussian fit parameters derived from diffuse reflection simulations and skin structure parameters: (**a**) Amplitude ($A$) versus SMT structure (${u}_{w}/{u}_{h}$), with ${W}_{V}/W$ fixed at 0.11. Inset: A versus sulcus cutis structure (${W}_{V}/W$), with ${u}_{w}/{u}_{h}$ fixed at 25. (**b**) Inverse of width ($1/\sigma$) versus SMT structure (${u}_{w}/{u}_{h}$), with ${W}_{V}/W$ fixed at 0.11. Inset: $1/\sigma$ versus sulcus cutis structure (${W}_{V}/W$), with ${u}_{w}/{u}_{h}$ fixed at 25. All simulations were performed with a fixed incident angle (${\theta }_{i}$) of 45°.

Figure 6 demonstrates the influence of SMT parameters on diffuse reflection (${\theta }_{i}$ is fixed at 45°), quantified by Gaussian fit parameters $A$ and $\sigma$. Figure 6(a) and 6(b) reveal linear relationships between $A$, $1/\sigma$ , and SMT structure (${u}_{w}/{u}_{h}$), confirming that lower ${u}_{w}/{u}_{h}$ values correspond to higher diffusion levels. The effect of microstructure, primarily captured by ${W}_{v}/W$, is illustrated in the insets of Fig. 6(a) and 6(b). While $A$ shows a linear inverse relationship with ${W}_{v}/W$, and $\sigma$ a linear positive correlation, the influence is less pronounced compared to ${u}_{w}/{u}_{h}$. Within the parameter ranges of interest (

Table 1), $A$ and $\sigma$ can be approximated by a fitted function of ${W}_{v}/W$ and ${u}_{w}/{u}_{h}$ (dashed lines in Fig. 6), expressed as:

2 $$A={I}_{s}\left[\left(0.0023-0.0016{W}_{v}/W\right){u}_{w}/{u}_{h}-0.00029\right]$$

3 $$\sigma ={\left[\left(0.006-0.0007{W}_{v}/W\right){u}_{w}/{u}_{h}-0.009\right]}^{-1}$$

where ${I}_{s}$ denotes the total scattering intensity.

**Fig. 7:**
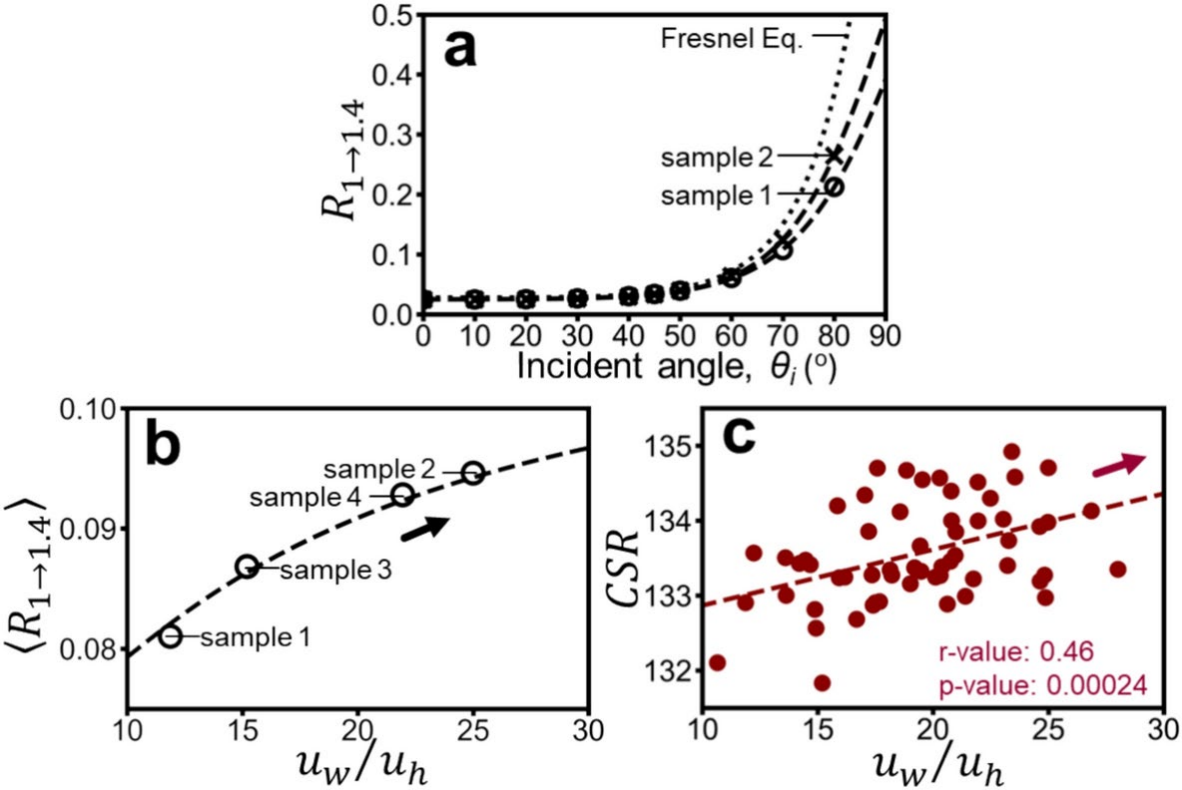
(**a**) Reflectance of diffuse reflection as a function of incident angle for sample 1 and sample 2. Circles and crosses represent simulation results for the soft and rough skin structures, respectively. Dashed lines show fitted trends using **Eq. **4, while the dotted line indicates reflectance for a flat surface (Fresnel equation). (**b**) Relationship between average reflectance (averaged over 0° to 90° incident angles using **Eq. **4) and ${u}_{w}/{u}_{h}$, demonstrating an increasing trend. (**c**) For comparison, the positive correlation between measured Crista cutis Surface Reflection (CSR) and ${u}_{w}/{u}_{h}$from a previous study^20^ is shown.

Figure 7 investigates the reflectance of diffuse reflection (${R}_{1\to 1.4}$) as a key indicator. Figure 7(a) shows its inverse relationship with SMT roughness (${u}_{w}/{u}_{h}$) across various incident angles (${\theta }_{i}$) for 2 SMT structures (soft: sample 1, rough: sample 2) and a perfectly flat surface. Notably, the trends for sample 3 and sample 4 lie between those of sample 1 and sample 2 but are omitted from the figure for clarity. The influence of ${W}_{v}/W$ is negligible, as previously established. The relationship is fitted with a sigmoid-like function (dashed lines):

4 $${R}_{1\to 1.4}\left({\theta }_{i},{u}_{w}/{u}_{h}\right)=1-\frac{0.00016+0.97/\left({u}_{w}/{u}_{h}\right)}{1+{e}^{\left(0.11-0.26/\left({u}_{w}/{u}_{h}\right)\right)\left[{\theta }_{i}-\left(87.44+107.15/\left({u}_{w}/{u}_{h}\right)\right)\right]}}$$

This function is derived from experimental data of 4 representative skin samples (${u}_{w}/{u}_{h}=$ 15.2, 21.9, 11.8, 25 with fixed ${W}_{v}=40$). Assuming uniform incident light intensity across all angles, the average reflectance as a function of ${u}_{w}/{u}_{h}$ is depicted in Fig. 7(b). It demonstrates that average reflectance increases with ${u}_{w}/{u}_{h}$, rising from 0.081 to 0.094 as ${u}_{w}/{u}_{h}$transitions from 11.8 to 25. This positive correlation aligns with the trend observed in^20^ between SMT Surface Reflection (CSR) and ${u}_{w}/{u}_{h}$ (Fig. 7(c)), although the measurement data does not exhibit a clear functional relationship.

## Diffuse transmission from skin subvisible micro texture

**Fig. 8:**
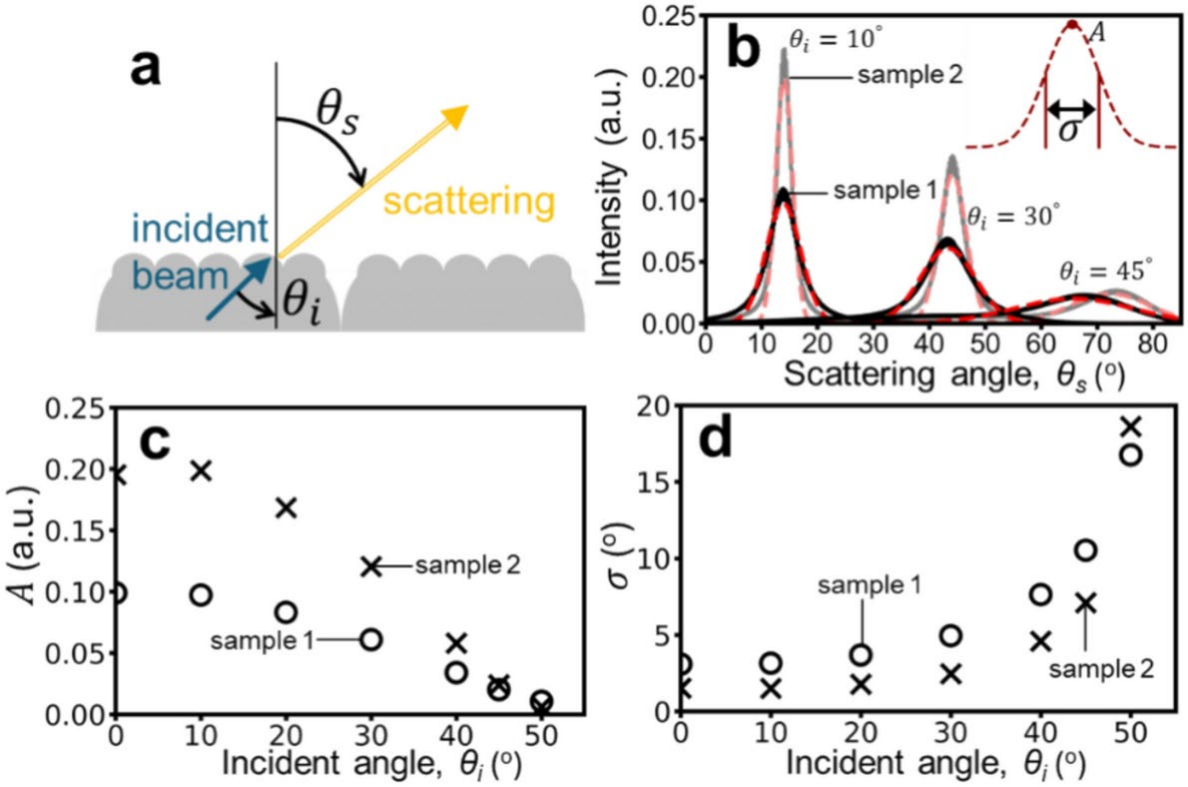
(**a**) Schematic illustration of the diffuse transmission process. (**b**) Comparison of scattering intensity distributions versus scattering angle for sample 1 (black) and sample 2 (gray). Solid lines represent simulated results, while dashed lines represent Gaussian fits. (**c**) Relationship between the Gaussian fit coefficient $A$ (intensity) and ${\theta }_{i}$(incident angle). (**d**) Relationship between the Gaussian fit coefficient $\sigma$ (width) and ${\theta }_{i}$(incident angle).

Figure 8(a) illustrates the physical process of diffuse transmission, where light within the skin undergoes diffuse scattering upon traversing the SMT. We investigated the scattering behavior for sample 1 and sample 2 under various incident angles (Fig. 8(b)). Due to the transition between different media, the central scattering angle shifts towards larger angles according to Snell’s law. Notably, sample 1 consistently exhibits a higher diffusion level (smaller $A$ and larger $\sigma$) than sample 2. Figures 8(c) and (d) record the changes in Gaussian fit parameters $A$ (amplitude) and $\sigma$ (width) for different ${\theta }_{i}$ (incident angles). Overall, sample 1 has a smaller $A$ and a larger $\sigma$. $A$ (amplitude) decreases with increasing ${\theta }_{i}$(incident angle), while $\sigma$ increases. Notably, the incident angle of 45° is close to the critical angle for a flat surface, but the SMT structure effectively increases this critical angle.

Applying the same methodology used for diffuse reflection, we derive the relationships between diffuse transmission parameters $A$ (amplitude) and $\sigma$ (width) and SMT parameters (${u}_{w}/{u}_{h}$ and ${W}_{v}/W$):

5 $$A={I}_{s}\left(1-{W}_{v}/W\right)\left[\left(0.028cos{\theta }_{i}-0.0196\right){u}_{w}/{u}_{h}+0.0493\left(1-cos{\theta }_{i}\right)\right]$$

6 $$\sigma ={\left\{\left(1-{0.71W}_{v}/W\right)\left[\left(0.089cos{\theta }_{i}-0.0604\right){u}_{w}/{u}_{h}+0.23\left(1-cos{\theta }_{i}\right)\right]\right\}}^{-1}$$

Note the additional $cos{\theta }_{i}$ term in these equations compared to **Eqs. **2 and 3, due to transmission through different media. Detailed results, like those in Fig. 6, are provided in the Appendix for reference.

**Fig. 9:**
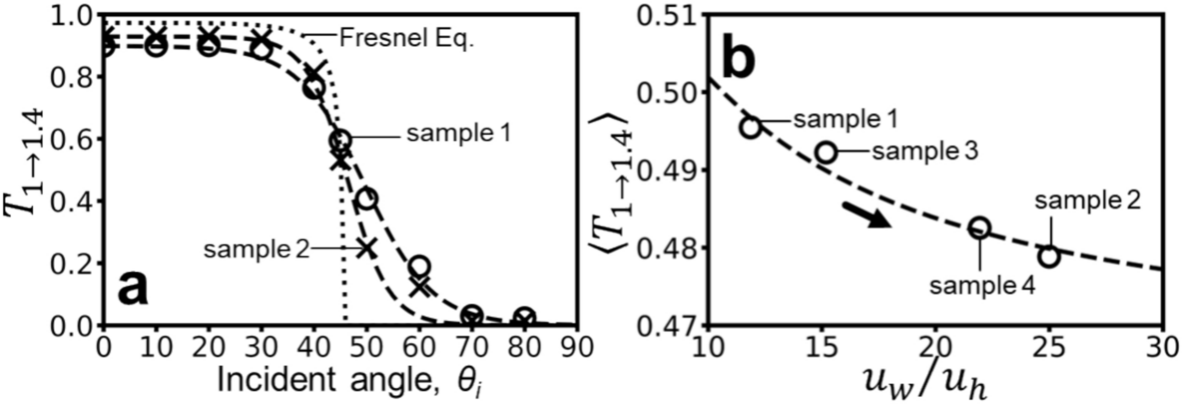
(**a**) Transmittance of diffuse transmission as a function of incident angle for sample 1 and sample 2. Circles and crosses represent simulation results for the soft and rough skin structures, respectively. Dashed lines show fitted trends using **Eq. **7, while the dotted line indicates transmittance for a flat surface (Fresnel equation). (**b**) Relationship between average reflectance (averaged over 0° to 90° incident angles using **Eq. **7) and ${u}_{w}/{u}_{h}$, demonstrating a decreasing trend.

Following the analysis of diffuse reflection, we investigate diffuse transmission in Fig. 9, using the same SMT parameters as in Fig. 7. Figure 9(a) shows the relationship between ${T}_{1.4\to 1}$ (transmittance) and ${\theta }_{i}$ (incident angle) for light propagating from skin to air, for both soft (circles) and rough (crosses) skin structures (sample 1 and 2). Data for samples 3 and 4, exhibiting intermediate trends, are omitted for clarity. Transmittance for a flat surface (Fresnel equation) is included as a reference. A sigmoid function quantitatively describes this trend:

7 $${T}_{1.4\to 1}\left({\theta }_{i},{u}_{w}/{u}_{h}\right)=\frac{0.94-0.44/\left({u}_{w}/{u}_{h}\right)}{1+{e}^{\left(0.32-2.00/\left({u}_{w}/{u}_{h}\right)\right)\left[{\theta }_{i}-\left(44.28+61.57/\left({u}_{w}/{u}_{h}\right)\right)\right]}}$$

The relationship between transmittance (${T}_{1.4\to 1}$), incident angle (${\theta }_{i}$), and ${u}_{w}/{u}_{h}$ is derived from fitting the SMT parameters from samples 1 to 4 (same used in Fig. 7(b)). Assuming a uniform distribution of incident light intensity across all angles (0° to 90°) from inner skin, the average transmittance as a function of ${u}_{w}/{u}_{h}$ is depicted in Fig. 9(b). As ${u}_{w}/{u}_{h}$ increases, the average transmittance (${\langle {T}_{1.4\to 1}\rangle }_{{0}^{^\circ }\le {\theta }_{i}\le {90}^{^\circ }}$) gradually decreases. When ${u}_{w}/{u}_{h}$ transitions from 11.8 to 25, (${\langle {T}_{1.4\to 1}\rangle }_{{0}^{^\circ }\le {\theta }_{i}\le {90}^{^\circ }}$) decreases from 0.495 to 0.479.

By deriving both the SMT-induced reflectance (**Eq. **4) and transmittance (**Eq. **7), we can further investigate the impact of SMT on translucency. We introduce a translucency index as an evaluation metric, defined as the proportion of internal skin reflections that penetrate the SMT and reach the skin surface. Directly proportional to translucency, this index quantitatively describes their relationship and comprises both direct reflection and diffuse scattering components, expressed as:

8 $${\tau }_{direct}={\langle \left[1-{R}_{1\to 1.4}\left({\theta }_{i},{u}_{w}/{u}_{h}\right)\right]{T}_{1.4\to 1}\left({\theta }_{i}{\prime},{u}_{w}/{u}_{h}\right)\rangle }_{0\le {\theta }_{i}\le {90}^{^\circ }}$$

9 $${\tau }_{diffuse}={\langle 1-{R}_{1\to 1.4}\left({\theta }_{i},{u}_{w}/{u}_{h}\right)\rangle }_{0\le {\theta }_{i}\le {90}^{^\circ }}{\langle {T}_{1.4\to 1}\left({\theta }_{i},{u}_{w}/{u}_{h}\right)\rangle }_{0\le {\theta }_{i}\le {90}^{^\circ }}$$

10 $$\tau ={\eta {\tau }_{diffuse}+\left(1-\eta \right)\tau }_{direct}$$

where ${\theta }_{i}{\prime}$ is the angle of refraction, for light traveling through different media, follows Snell’s law. $\eta$ is the ratio of diffuse component to total energy of diffusive transmission. $\tau$ denotes total translucency index.

**Fig. 10:**
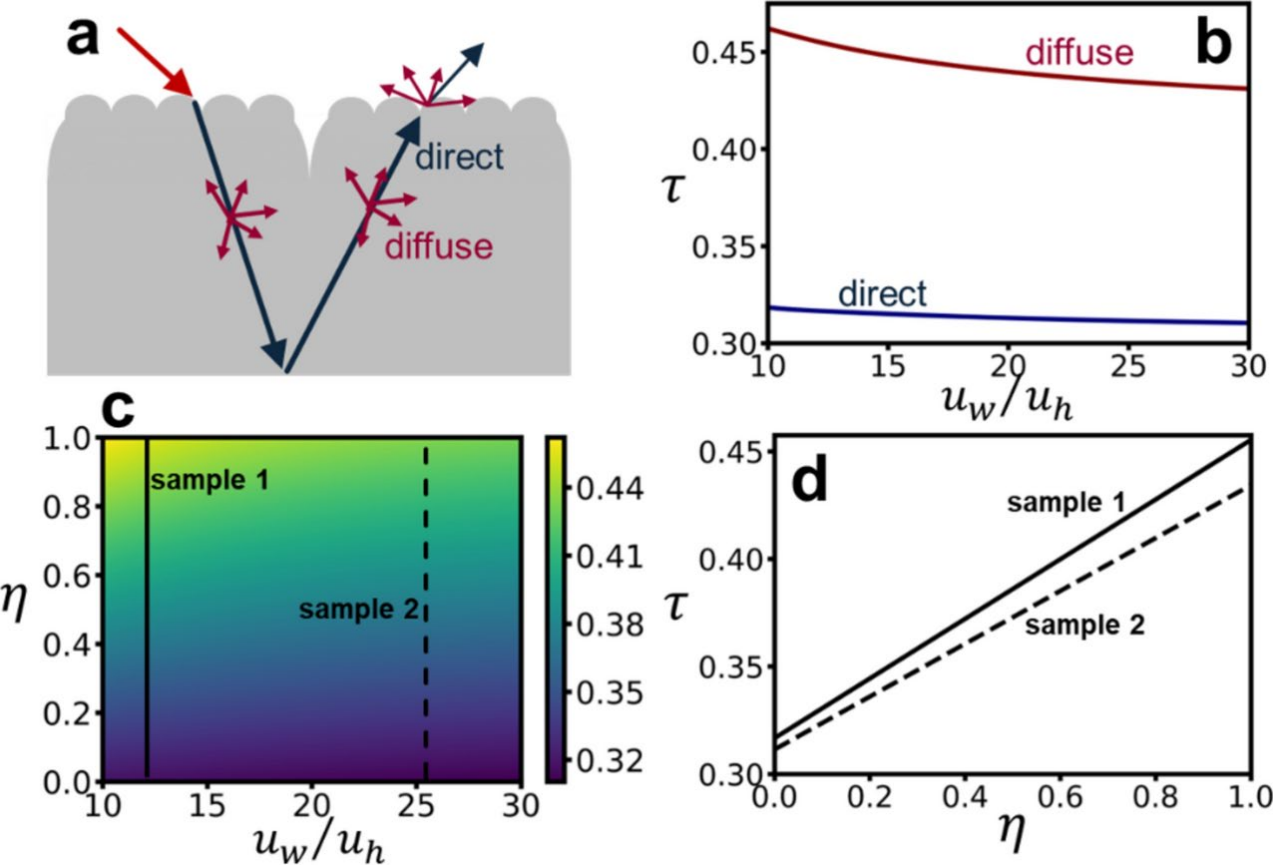
summarizes the relationship between the parameter ${u}_{h}/{u}_{w}$ and translucency: (**a**) a schematic description of translucency due to skin internal reflection, divided into direct and diffuse reflection components. (**b**) The translucency index ($\tau$) contributed by direct and diffuse reflection components (refer to **Eqs. **8 and 9) can be expressed as a function of the parameter ${u}_{h}/{u}_{w}$. (**c**) Changes in the translucency index and ${u}_{h}/{u}_{w}$ when altering the ratio of diffuse ($\eta$). The solid line represents sample 1, while the dashed line represents sample 2. (**d**) Following (**c**), the relationship between the translucency index ($\tau$) and the ratio of diffuse ($\eta$) by comparing sample 1 and sample 2.

We consider isotropic incident light (equal energy in all directions) penetrating the skin, which then converts into direct reflection and diffuse scattering components within the skin. The direct reflection component maintains the original incident direction, undergoes complete reflection by the next skin layer, and then diffusely transmits through the SMT. The diffuse scattering component undergoes diffuse transmission through the SMT in all directions within the skin. Figure 10(b) shows the translucency indices for direct reflection and diffuse scattering. When ${u}_{w}/{u}_{h}$ is smaller, $\tau$ is higher, with the diffuse scattering translucency index (${\tau }_{diffuse}$) exceeding that of direct reflection (${\tau }_{direct}$). Figure 10(c) illustrates the total translucency index ($\tau$) under conditions with different proportions of diffuse scattering in internal reflection. It is evident that when ${u}_{w}/{u}_{h}$ is smaller and there is a higher proportion of diffuse scattering ($\eta$), the overall translucency index is larger. In Fig. 10(c), we also indicate the positions of Sample 1 and Sample 2 in the parameter space. The nearly linear variation of the translucency index ($\tau$) and the ratio of diffuse scattering ($\eta$) is shown in Fig. 10(d).

## Discussion

The influence of subvisible micro texture (SMT) on light scattering behavior is a complex interplay of two structures of different scales: the SMT and the macro texture. To dissect this relationship, we employed SMT scans and parameterized an equivalent 2D SMT model for numerical simulation.

Initial validation of this model involved comparing simulated and measured light scattering (diffuse reflection and transmission) from PMMA samples (HD6 and SB6), materials commonly employed to mimic the scattering effects of cosmetics and skin^9,21^, with a Gonio Photometer GP-700 (Fig. 4). The strong agreement in scattering angle distributions and peak shifts affirmed the model’s accuracy.

Subsequently, we applied this model to real skin SMT, utilizing data from a previous study on young Asian female cheek skin (Fig. 5). These samples, classified as “soft” or “rough” based on visual assessment, were previously linked to diffuse reflection levels^20^. By quantifying diffuse scattering patterns with Gaussian fitting, we confirmed that softer skin consistently exhibited higher diffusion (lower $A$, higher $\sigma$), regardless of incident light direction.

Spearman’s correlation analysis (Table 2) pinpointed ${u}_{w}/{u}_{h}$ (for SMT) and ${W}_{v}/W$ (for macro texture) as the dominant SMT parameters influencing $A$ and $\sigma$. Notably, ${u}_{w}/{u}_{h}$ exhibited a stronger correlation than ${W}_{v}/W$. This was further explored in Fig. 6, where simulations within relevant parameter ranges (Table 1) revealed a clear linear relationship between ${u}_{w}/{u}_{h}$ and both $A$ and $\sigma$, with lower ${u}_{w}/{u}_{h}$ values corresponding to increased diffusion. While ${W}_{v}/W$ also influenced $A$ and $\sigma$, the effect was significantly weaker, consistent with the correlation analysis. These relationships were then consolidated into empirical equations (**Eqs. **2 and 3), providing a quantitative link between SMT parameters and scattering behavior. The inverse relationship between ${u}_{w}/{u}_{h}$and diffusion level aligns with prior research^22^, highlighting the role of surface gradient variations in light scattering.

To further explore the practical implications of these findings, we investigated SMT reflectance. Figure 7 presents simulations for four representative skin samples, demonstrating that reflectance as a function of incident angle can be effectively modeled using a sigmoid-like function dependent on ${u}_{w}/{u}_{h}$. Assuming isotropic incident light, we observed a positive correlation between average reflectance and ${u}_{w}/{u}_{h}$, mirroring previous findings on Crista cutis Surface Reflection (CSR)^20^.

These results collectively offer quantitative insights into how SMT modulates the soft-focus effect, where light diffusion minimizes the appearance of imperfections. **Equations **4, 5, and 6 elucidate the optimal SMT structures for maximizing this effect. However, it is crucial to note that these empirical formulas are valid only within the parameter ranges specified in Table 1, underscoring the need for further research on extreme SMT conditions.

The influence of SMT extends to diffuse transmission, as illustrated in Fig. 8. While refraction effects are present due to the change in medium, the relationship between scattering and SMT parameters remains consistent with that of diffuse reflection. Figure 9 and **Eqs. **7 and 8 summarize these correlations, confirming the dominance of ${u}_{w}/{u}_{h}$ over ${W}_{v}/W$ in influencing scattering. The average transmittance, under isotropic incident light, exhibits a negative correlation with ${u}_{w}/{u}_{h}$.

Finally, Fig. 10delves into the impact of SMT on skin translucency, a complex optical property with varying definitions^23–29^. Our proposed translucency index, positively correlated to translucency, allows for a quantitative assessment of this relationship. By separating inner reflection into direct and diffuse components, we find that skin translucency is enhanced when ${u}_{w}/{u}_{h}$ is small and the diffuse component is large (see Fig. 10(c)). These findings align with existing experimental observations and support previous research on skin optical properties. Additionally, in Fig. 10(d), we note the variation in the translucency index ($\tau$) for sample 1 and sample 2 under different proportions of the diffuse component ($\eta$), with the former being greater than the latter.

It is important to acknowledge that our focus is on the SMT, using a simplified single-layer skin model (see Fig. 10** (a)**). While the internal mechanisms converting incident light into direct and diffuse components are not explicitly modeled, our comprehensive exploration of how varying proportions of these components affect the translucency index allows for a rapid and reliable estimation of skin translucency across a wide range of scenarios. However, unlike other studies that define translucency as the ratio of diffuse reflected light to incident light, accounting for scattering across multiple skin layers, our model focuses solely on the stratum corneum with isotropic scattering assumptions. Future advancements could integrate multilayer Monte Carlo simulations and detailed biological data on chromophores like melanin and hemoglobin to provide a more accurate representation of skin translucency and its dependence on both SMT and internal structures.

## Conclusion

This study has unraveled the intricate relationship between skin macrotexture (SMT) and light scattering behavior, including diffuse reflection and transmission, revealing the critical role of the SMT and macro texture in modulating the soft-focus effect and skin translucency. By employing SMT scans and parameterizing an equivalent 2D SMT model, we successfully simulated light scattering patterns and validated our approach using PMMA samples and real skin data.

The SMT parameter ${u}_{w}/{u}_{h}$ and the macro texture parameter ${W}_{v}/W$ were identified as the primary determinants of light scattering behavior, with ${u}_{w}/{u}_{h}$ exhibiting a more pronounced influence. Our findings consistently demonstrate that softer skin, characterized by a lower ${u}_{w}/{u}_{h}$, exhibits higher diffusion levels, regardless of incident light direction. This trend was observed in both diffuse reflection and transmission, where softer skin consistently exhibited lower $A$ and higher $\sigma$ in the scattering patterns. In the case of diffuse reflection, a lower ${u}_{w}/{u}_{h}$, results in a higher level of diffusion, directly translating to a more pronounced soft-focus effect. This observation is consistent with previous studies^20,22^.

Additionally, we explored the impact of SMT on skin translucency, a complex optical property. Our proposed translucency index allowed for a quantitative assessment of this relationship. We found that skin translucency is enhanced when ${u}_{w}/{u}_{h}$​​ is small and the diffuse component is large. These findings align with existing experimental observations and support previous research on skin optical properties.

This study establishes a quantitative framework linking SMT parameters to light scattering behavior, revealing how SMT affects the soft-focus effect and skin translucency. Our findings, supported by the diffuse transmission imaging system (DTIS), offer valuable insights into the development of cosmetics, skincare products, and applications in aesthetic medical imaging and dermatology^30^. The DTIS methodology enables precise evaluation of skin texture improvements from cosmetic procedures such as intense pulsed light (IPL) treatments, providing objective measurements of changes in SMT parameters^31^. While our simplified equivalent 2D SMT model provides a solid understanding of skin translucency within specific parameter ranges, future research should incorporate multilayered skin models to account for the complex optical properties of different skin layers and the effects of chromophores such as melanin and hemoglobin. This would enable a more nuanced understanding of light-skin interactions, particularly under extreme SMT conditions, and improve the accuracy of simulations. Additionally, investigating the impact of anisotropic microstructures on light scattering and visual appearance^32,33^ could further enhance our understanding of skin optical properties. Combining these advancements with comparisons to other measurement techniques would not only validate our translucency index but also broaden its applicability in fields such as cosmetics, aesthetic medicine, and computer graphics, where precise representation of skin optics is essential.

## Supplementary Information

Supplementary Information.

## Data Availability

The datasets used and/or analysed during the current study available from the corresponding author on reasonable request.
