# Supplementary material for: Study of diffuse scattering on facial surface using ray tracing approach
Source: Sci Rep. 2025 Apr 12;15:12597. doi: 10.1038/s41598-025-89113-x (PMC11993760; doi:10.1038/s41598-025-89113-x)
Supplement: Supplementary file 1 — Supplementary Information. [file 41598_2025_89113_MOESM1_ESM.docx]

**Appendix**

**Figure 8(b)** illustrates the angular distribution of diffuse transmission intensity for different skin samples. Like backscattering, the smaller the ${u_{w}}/{u_{h}}$, the greater the diffusion level of scattering. However, a key distinction lies in the offset of the scattering center position towards larger angles due to the propagation of incident and scattered light in different media. **Figure 8(c)** and **Figure 8(d)** delve into the relationship between the diffusion level parameters $A$ and $\sigma$ and the ${u_{w}}/{u_{h}}$. **Figure 8(c)** reveals an inverse relationship between $A$ and ${u_{w}}/{u_{h}}$, indicating that $A$ decreases as ${u_{w}}/{u_{h}}$ increases. In contrast, **Figure 10(c)** demonstrates a positive correlation between $\sigma$ and ${u_{w}}/{u_{h}}$, signifying that $\sigma$ increases with increasing ${u_{w}}/{u_{h}}$.

**Figure 8(c)** and **Figure 8(d)** presented the relationships between the diffusion level parameters $A$ and $\sigma$ and the ${u_{w}}/{u_{h}}$. To further explore these relationships, the trends can be plotted against the cosine of the incident angle ($cos\theta_{i}$) in **Appendix figure 1(a)** and **Appendix figure 1(b)**. This indicates that $A$ and $1/\sigma$ increases linearly with increasing $cos\theta_{i}$. The linear relationships between $A$ and $\sigma$ with $cos\theta_{i}$ can be summarized as follows:

$A=\alpha_{1}cos\theta_{i}+\beta_{1}$, (11)

$\sigma=\left( \alpha_{2}cos\theta_{i}+\beta_{2} \right)^{-1}$. (12)

**Equation 11** and **Equation 12** introduced the parameters $\alpha_{1}$, $\alpha_{2}$, $\beta_{1}$, and $\beta_{2}$, which play a crucial role in characterizing the scattering behavior of light in skin. To investigate the relationship between these parameters and skin structural characteristics, we examined their dependence on the SMT structural parameter ${u_{w}}/{u_{h}}$ and the macrostructural parameter ${W_{v}}/W$ within the relevant parameter range. **Appendix figure 2** reveals a linear positive correlation between the parameters $\alpha_{1}$ and $\alpha_{2}$ and the SMT structural parameter ${u_{w}}/{u_{h}}$. This suggests that as ${u_{w}}/{u_{h}}$ increases, the values of $\alpha_{1}$ (see **Appendix figure 2(a)**) and $\alpha_{2}$ (see **Appendix figure 2(c)**) also increase. Conversely, the parameters $\beta_{1}$ (see **Appendix figure 2(b)**) and $\beta_{2}$ (see  **Appendix figure 2(d)**) exhibit a linear inverse relationship with ${u_{w}}/{u_{h}}$. This implies that $\beta_{1}$ and $\beta_{2}$ decrease as ${u_{w}}/{u_{h}}$ increases. **Appendix figure 3** shows that both $A$ (see **Appendix figure 3(a)**) and $1/\sigma$ (see **Appendix figure 3 (b)**) have a linear relationship with ${W_{v}}/W$. As ${W_{v}}/W$ increases, $A$ also increases, while σ decreases. This trend is like what we observed in **Figure 8**. The dashed lines in **Appendix figure 2** and **Appendix figure 3** represent fitting relationships derived from **Equation 5** and **Equation 6**, which exhibit high consistency with the simulation results of forward scattering diffusion parameters ($A$ and $\sigma$) within the specified parameter range.


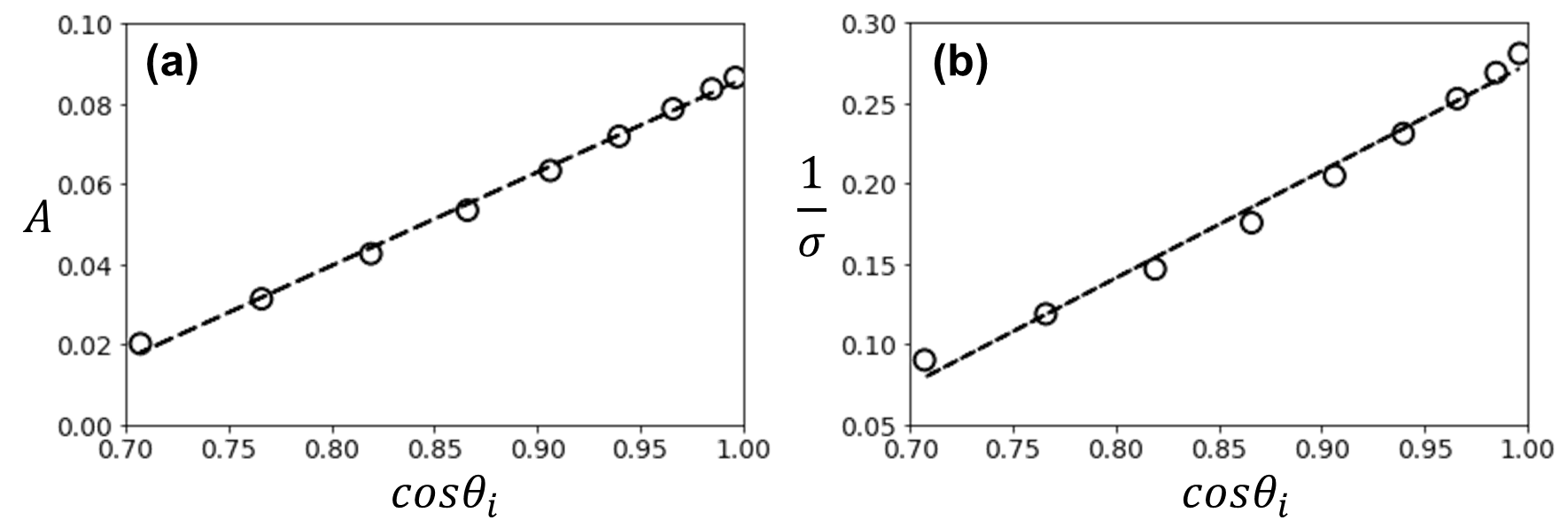


**Appendix figure 1.** The Gaussian fitting parameters, **(a)**$A$ and **(b)** $1/\sigma$, obtained from forward scattering simulations exhibit a linear relationship with $cos\theta_{i}$. This relationship is observed under fixed ${u_{h}}/{u_{w}=15}$ and ${W_{V}}/W=0.12$ conditions.


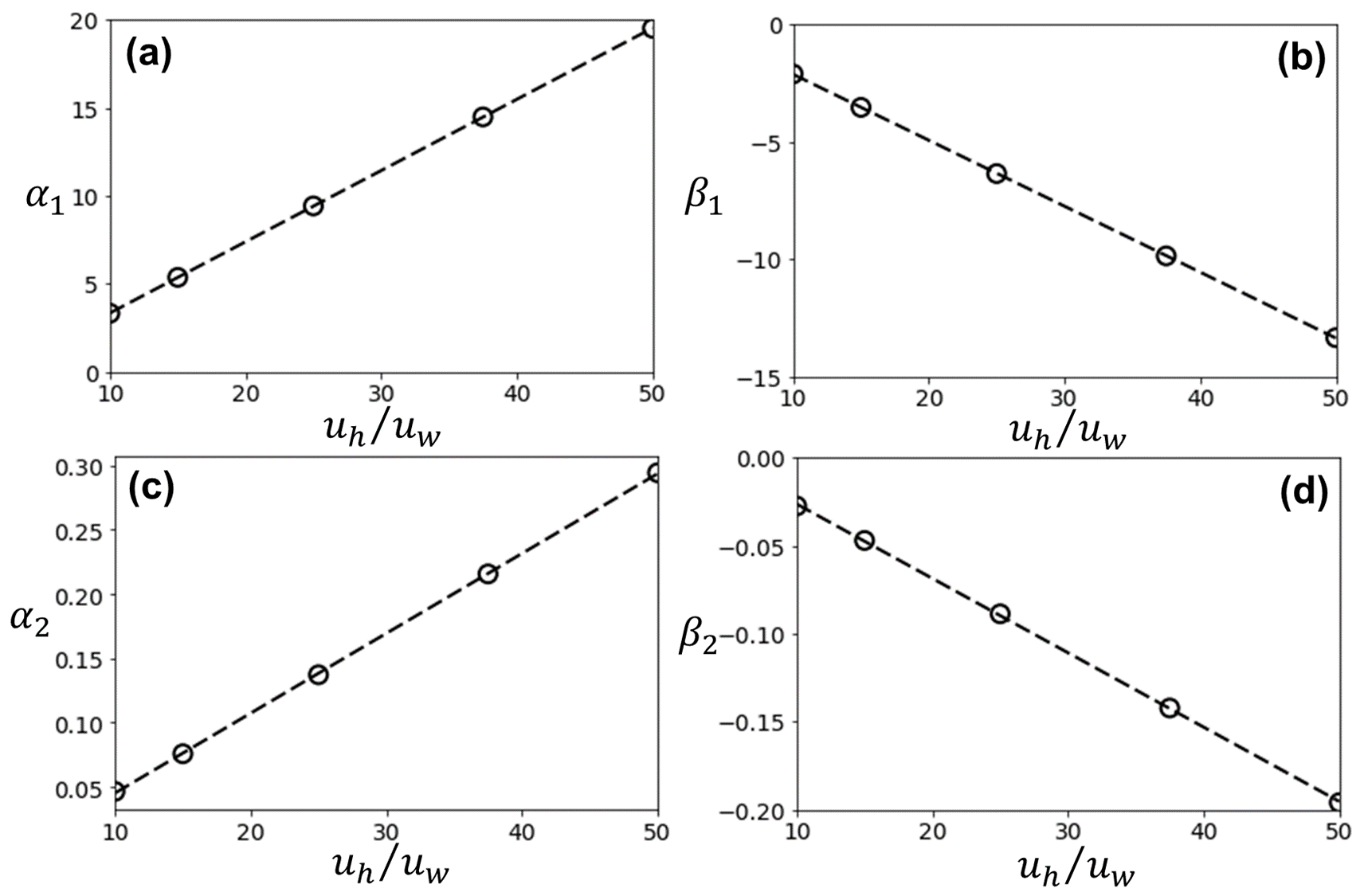


**Appendix figure 2.** The linear relationships depicted in **Equation 6** and **Equation 7** can be further analyzed to reveal a linear dependence of the fitting parameter coefficients on the ${u_{h}}/{u_{w}}$ (with ${W_{V}}/W$ fixed at 0.12). Panels **(a)**, **(b)**, **(c)**, and **(d)** illustrate the linear relationships between $\alpha_{1}$, $\beta_{1}$, $\alpha_{2}$, and $\beta_{2}$, respectively, and ${u_{h}}/{u_{w}}$.


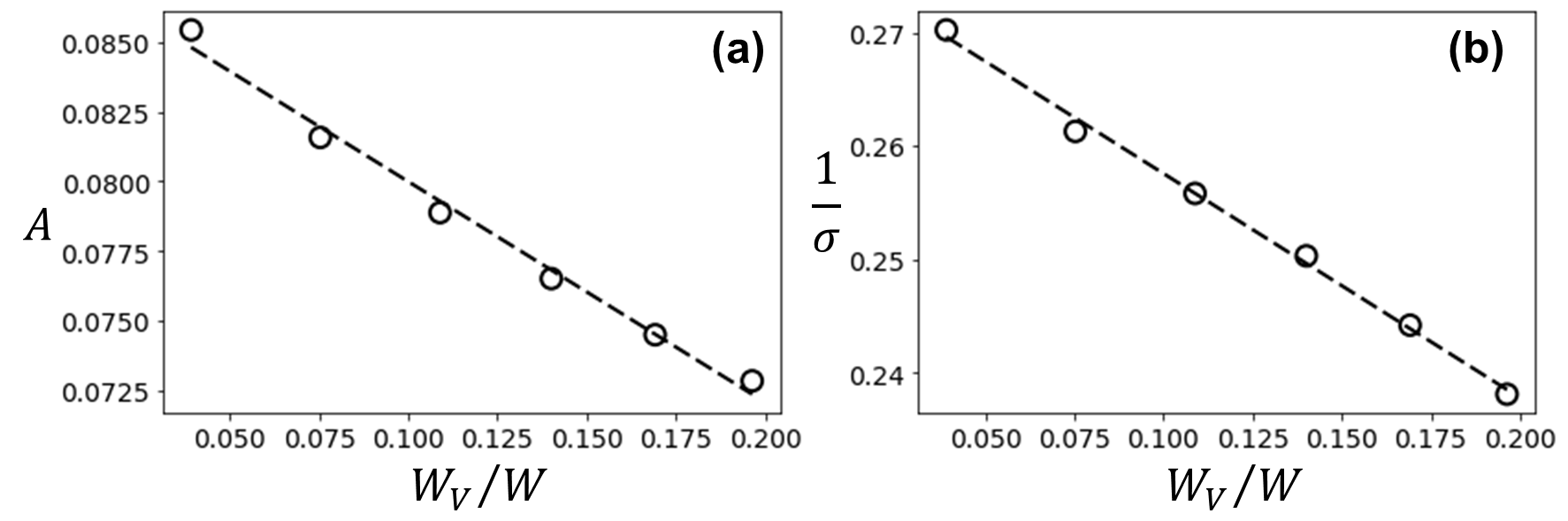


**Appendix figure 3.** Linear relationship between the fitting parameters: **(a)** $A$ and **(b)** $1/\sigma$ obtained from Gaussian fitting of diffuse transmission simulation results and the structure of the sulcus cutis, parameterized by ${W_{V}}/W$ (with fixed ${u_{w}}/{u_{h}}$ at 10).
